# The Impact of Ferric Derisomaltose on Cardiovascular and Noncardiovascular Events in Patients With Anemia, Iron Deficiency, and Heart Failure With Reduced Ejection Fraction

**DOI:** 10.1016/j.cardfail.2023.10.006

**Published:** 2024-05

**Authors:** Robin Ray, Ian Ford, John G.F. Cleland, Fraser Graham, Fozia Z. Ahmed, Abdallah Al-Mohammad, Peter J. Cowburn, Chris Critoph, Philip A. Kalra, Rebecca E. Lane, Andrew Ludman, Pierpaolo Pellicori, Mark C. Petrie, Michelle Robertson, Alison Seed, Iain Squire, Paul R. Kalra, INVESTIGATORS, Paul Kalra, Elena Cowan, Serena Howe, Charlotte Turner, Rosalynn Austin, Rebeca Lane, Paula Rogers, Paul Foley, Badri Chandrasekaran, Eva Fraile, Lynsey Kyeremeh, Fozia Ahmed, Mark Petrie, Lorraine McGregor, Joanna Osmanska, Fraser Graham, Ninian Lang, Barbara Meyer, Faheem Ahmad, Joanna Osmanska, Iain Squire, Jude Fisher, Philip Kalra, Christina Summersgill, Katarzyna Adeniji, Rajkumar Chinnadurai, Andrew Ludman, Lisa Massimo, Clare Hardman, Daisy Sykes, Peter Cowburn, Sarah Frank, Simon Smith, Alan Japp, Mohamed Anwar, Beth Whittington, Alison Seed, Robin Ray, Vennessa Sookhoo, Abdallah Al-Mohammad, Janet Middle, Kay Housley, Andrew Clark, Jeanne Bulemfu, Christopher Critoph, Victor Chong, Stephen Wood, Benjamin Szwejkowski, Chim Lang, Jackie Duff, Susan MacDonald, Rebekah Schiff, Patrick Donnelly, Thuraia Nageh, Swapna Kunhunny, Mark Petrie, Roy Gardner, Marion McAdam, Elizabeth McPherson, Prithwish Banerjee, Eleanor Sear, Nigel Edwards, Jason Glover, Pierpaolo Pellicori, Clare Murphy, Justin Cooke, Charles Spencer, Mark Francis, Iain Matthews, Hayley McKie, Andrew Marshall, Janet Large, Jenny Stratford, Piers Clifford, Sara Tavares, Christopher Boos, Philip Keeling, Debbie Hughes, Aaron Wong, Deborah Jones, Alex James, Rhys Williams, Stephen Leslie, Jim Finlayson, Piers Clifford, Andrew Hannah, Philip Campbell, John Walsh, Jane Quinn, Callum Chapman, Susan Piper, Preeti Gupta, Victor Sim, Lucy Knibbs, Kristopher Lyons, Lana Dixon, Colin Petrie, Yuk-ki Wong, Catherine Labinjoh, Simon Duckett, Ian Massey, Henry Savage, Sofia Matias, Jonaifah Ramirez, Charlotte Manisty, Ifza Hussain, Rajiv Sankaranarayanan, Gershan Davis, Samuel McClure, John Baxter, Eleanor Wicks, Jolanta Sobolewska, Jerry Murphy, Ahmed Elzayat, Alastair Cooke, Jay Wright, Simon Williams, Amal Muthumala, Parminder Chaggar, Gethin Ellis, Mandie Welch, Sudantha Bulugahapitiya, Thomas Jackson, Tapesh Pakrashi, Ameet Bakhai, Reto Gamma, Susan Ellery, Charlotte Manisty, Geraint Jenkins, Angus Nightingale, CLINICAL TRIALS UNIT AND DATA AND STATISTICAL CENTRE, Elizabeth Thomson, Ian Ford, Michele Robertson, Nicola Greenlaw, Kirsty Wetherall, Ross Clarke, Christopher Graham, Sharon Kean, Alan Stevenson, Robbie Wilson, Sarah Boyle, John McHugh, Lisa Hall, Joanne Woollard, Claire Brunton, Eleanor Dinnett, Amanda Reid, NURSING AND ADMINISTRATIVE SUPPORT, Jill Nicholls, Anna Cunnington, Serena Howe, SPONSOR SUPPORT, Elizabeth Douglas, Margaret Fegen, Marc Jones, Sheila McGowan, Barbara Ross, Pamela Sandu, Pamela Surtees, Debra Stuart

**Affiliations:** Queen Alexandra Hospital, Portsmouth, UK; Royal Brompton and Harefield Hospital, London, UK; Great Western Hospital, Swindon, UK; Manchester Royal Infirmary, Manchester, UK; Glasgow Royal Infirmary, Glasgow, UK; Queen Elizabeth University Hospital, Glasgow, UK; Glenfield Hospital, Leicester, UK; Salford Royal Hospital, Salford, UK; Royal Devon and Exeter Hospital, Exeter, UK; University Hospital Southampton, Southampton, UK; Royal Infirmary of Edinburgh, Edinburgh, UK; Blackpool Victoria Hospital, Blackpool, UK; Sinead Lyons, St. George's Hospital, London, UK; Northern General Hospital, Sheffield, UK; Castle Hill Hospital, Hull, UK; Royal Bournemouth Hospital, Bournemouth, UK; University Hospital Crosshouse, Kilmarnock, UK; Ninewells Hospital, Dundee, UK; Guy's and St Thomas’ Hospital, London, UK; Ulster Hospital, Dundonald, UK; Southend University Hospital, Southend, UK; Golden Jubilee National Hospital, Clydebank, UK; University Hospital Coventry, Coventry, UK; Basingstoke and North Hampshire Hospital, Basingstoke, UK; Royal Alexandra Hospital, Paisley, UK; Chesterfield Royal Hospital, Chesterfield, UK; New Cross Hospital, Wolverhampton, UK; Victoria Hospital, Kirkcaldy, UK; Wansbeck General Hospital, UK; District General Hospital, Eastbourne, UK; Hammersmith Hospital, London, UK; Poole Hospital, Poole, UK; Torbay Hospital, Torquay, UK; Princess of Wales Hospital, Bridgend, UK; Raigmore Hospital, Inverness, UK; Wycombe Hospital, High Wycombe, UK; Aberdeen Royal Infirmary, Aberdeen, UK; Royal Gwent Hospital, Newport, UK; Nottingham University Hospital, Nottingham, UK; West Middlesex University Hospital, Isleworth, UK; Sheetal Patale, King's College Hospital, London, UK; University Hospital Llandough, Penarth, UK; Antrim Area Hospital, Antrim, UK; Royal Victoria Hospital, Belfast, UK; University Hospital Monklands, Airdrie, UK; St Richard's Hospital, Chichester, UK; Forth Valley Royal Hospital, Larbert, UK; Royal Stoke University Hospital, Stoke-On-Trent, UK; Basildon University Hospital, Basildon, UK; St Bartholomew's Hospital, London, UK; Aintree University Hospital, Liverpool, UK; Sunderland Royal Hospital, Sunderland, UK; John Radcliffe Hospital, Oxford, UK; Royal Oldham Hospital, Oldham, UK; Darlington Memorial Hospital, Darlington, UK; Doncaster Royal Infirmary, Doncaster, UK; Liverpool Heart and Chest Hospital, Liverpool, UK; Wythenshawe Hospital, Manchester, UK; North Middlesex University Hospital, London, UK; Sue Webber, Royal Cornwall Hospital, Truro, UK; Royal Glamorgan Hospital, Llantrisant, UK; Bradford Royal Infirmary, Bradford, UK; Salisbury District Hospital, Salisbury, UK; Kingston Hospital, Kingston Upon Thames, UK; Vinodh Krishnamurthy, Barnet Hospital, Barnet, UK; Broomfield Hospital, Chelmsford, UK; Royal Sussex County Hospital, Brighton, UK; University College London Hospital, London, UK; Gladdys Thomas, Morriston Hospital, Swansea, UK; Bristol Royal Infirmary, Bristol, UK; Robertson Centre for Biostatistics, University of Glasgow; NHS Tayside, Dundee, UK; Portsmouth Hospitals University NHS Trust, Portsmouth, UK; From NHS Greater Glasgow and Clyde, Glasgow, UK; From University of Glasgow, Glasgow, UK; 1 Department of Cardiology, St George's University Hospitals NHS Foundation Trust, London, UK; 2 Molecular and Clinical Sciences Institute, St. George's University of London, United; 3 Robertson Centre for Biostatistics, University of Glasgow, Glasgow, UK; 4 School of Cardiovascular and Metabolic Health, University of Glasgow, Glasgow, UK; 5 Department of Cardiology, Manchester University NHS Foundation Trust, Manchester, UK; 6 Department of Cardiology, Sheffield Teaching Hospitals NHS Foundation Trust, Sheffield, UK; 7 Department of Infection, Immunity and Cardiovascular Disease, The University of Sheffield, Sheffield, UK; 8 Department of Cardiology, University Hospital Southampton NHS Foundation Trust, Southampton, UK; 9 Department of Cardiology, University Hospitals Dorset NHS Foundation Trust, Bournemouth, UK; 10 Department of Renal Medicine, Northern Care Alliance NHS Foundation Trust Salford Care Organisation, Salford, UK; 11 Department of Cardiology, Royal Brompton and Harefield Hospitals, London, UK; 12 Department of Cardiology, Royal Devon University Healthcare NHS Foundation Trust, Exeter, UK; 13 Department of Cardiology, Blackpool Teaching Hospitals NHS Foundation Trust, Blackpool, UK; 14 Department of Cardiovascular Sciences, University of Leicester, Leicester, UK; 15 Department of Cardiology, Portsmouth Hospitals University NHS Trust, Portsmouth, UK; 16 College of Medical, Veterinary and Life Sciences, University of Glasgow, Glasgow, UK; 17 Faculty of Science and Health, University of Portsmouth, Portsmouth, UK

## Abstract

**Background:**

In some countries, intravenous ferric derisomaltose (FDI) is only licensed for treating iron deficiency with anemia. Accordingly, we investigated the effects of intravenous FDI in a subgroup of patients with anemia in the IRONMAN (Effectiveness of Intravenous (IV) Iron Treatment Versus Standard Care in Patients With Heart Failure and Iron Deficiency) trial.

**Method and Results:**

IRONMAN enrolled patients with heart failure, a left ventricular ejection fraction of ≤45%, and iron deficiency (ferritin <100 µg/L or transferrin saturation of <20%), 771 (68%) of whom had anemia (hemoglobin <12 g/dL for women and <13 g/dL for men). Patients were randomized, open label, to FDI (*n* = 397) or usual care (*n* = 374) and followed for a median of 2.6 years. The primary end point, recurrent hospitalization for heart failure and cardiovascular death, occurred less frequently for those assigned to FDI (rate ratio 0.78, 95% confidence interval 0.61–1.01; *P* = .063). First event analysis for cardiovascular death or hospitalization for heart failure, less affected by the coronavirus disease 2019 pandemic, gave similar results (hazard ratio 0.77, 95% confidence interval 0.62–0.96; *P* = .022). Patients randomized to FDI reported a better Minnesota Living with Heart Failure quality of life, for overall (*P* = .013) and physical domain (*P* = .00093) scores at 4 months.

**Conclusions:**

In patients with iron deficiency anemia and heart failure with reduced left ventricular ejection fraction, intravenous FDI improves quality of life and may decrease cardiovascular events.

## Highlights

• This analysis reports the effects of intravenous ferric derisomaltose (FDI) in a population of patients with heart failure and iron deficiency anemia in the IRONMAN trial.
• Intravenous FDI was well-tolerated and improved quality of life and may reduce morbidity and mortality in patients with heart failure, anemia, and iron deficiency.
• This will help shared decision-making in the management of patients with heart failure in countries where intravenous FDI is only licensed for the treatment of iron deficiency when accompanied by anemia.

## Graphical Abstract

**Figure:**
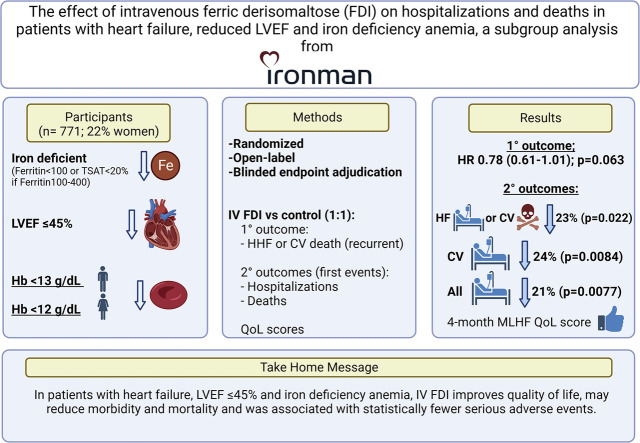

Iron deficiency is common in patients with heart failure and a reduced left ventricular ejection fraction (LVEF), and, when present, it is associated with greater impairment of quality of life and a higher risk of hospitalization for heart failure and cardiovascular death.^1^, ^2^, ^3^, ^4^ Iron deficiency is more common in patients with coexistent anemia.^5^

The IRONMAN (Effectiveness of Intravenous (IV) Iron Treatment Versus Standard Care in Patients With Heart Failure and Iron Deficiency) trial evaluated the impact of repeated doses of IV ferric derisomaltose (FDI) on the composite end point of recurrent heart failure hospitalizations and cardiovascular death in a broad range of patients with heart failure, reduced LVEF (≤45%), and iron deficiency.^6^ Patients were excluded if they had a hemoglobin of <9.0 g/dL. Men with a hemoglobin of ≤14 g/dL and women with hemoglobin of ≤13 g/dL could be enrolled; therefore, many patients did not have anemia.^7^

Overall, FDI decreased the primary end point, although this was not statistically significant (rate ratio for the primary analysis 0.82, 95% confidence interval [CI] 0.66–1.02, *P* = .07).^8^ Given that a sizeable proportion of the trial was conducted during the coronavirus disease 2019 (COVID-19) pandemic, with concerns that recurrent iron deficiency may not have been detected or corrected and noting the pandemic's impact on heart failure hospitalization, a prespecified COVID-19 sensitivity analysis was performed. This included all patients randomized until March 31, 2020, with follow-up censored on September 30, 2020 (see Methods), and showed a larger and significant reduction in the primary end point with a rate ratio of 0.76 (95% CI 0.58–1.00, *P* = .047).^8^

In some countries, IV FDI is licensed only for the treatment of iron deficiency when accompanied by anemia. Accordingly, we now report the effects of IV FDI in the population of patients with heart failure and iron deficiency anemia in the IRONMAN trial.

## Methods

The study design of IRONMAN, a prospective randomized open-label blinded-end point event-driven trial, has been described in detail.^6^^,^^8^ Briefly, patients aged ≥18 years with LVEF ≤45% and iron deficiency (ferritin <100 µg/L and/or transferrin saturation [TSAT] <20%, provided ferritin ≤400 µg/L) were invited to participate if they had a current or recent (within 6 months) hospitalization for heart failure or elevated natriuretic peptide plasma concentration. The full inclusion and exclusion criteria have been published.^6^ Patients were randomized to usual care alone (which permitted oral iron at investigators’ discretion but not IV iron) or IV FDI, the dose of which was calculated according to patients’ weight and hemoglobin to a maximum of 2000 mg per infusion.^6^ Randomization was stratified by recruitment context and trial site. Follow-up occurred at 4 weeks, 4 months, and then every 4 months, and, for patients randomized to IV FDI, further infusion was undertaken if either the ferritin was <100 µg/L or TSAT was <25% (provided ferritin ≤400 µg/L). At each study visit, investigators were encouraged to optimize other treatments directed toward heart failure in all patients according to contemporary guidelines. At 4 and 20 months, the Minnesota Living with Heart Failure (MLHFQ) questionnaire was recorded.

Patients were invited to consent to record linkage to national databases of deaths and hospital discharge summaries (National Health Service Digital and Public Health Scotland). These records were then used to highlight potential events to local investigators so that they could be reviewed and submitted per protocol, thereby maximizing the completeness of event recording. All hospitalizations and deaths were adjudicated blindly.

The analyses presented in this manuscript included only those patients who met the World Health Organization (WHO) criteria for anemia at randomization: women with a hemoglobin of <12 g/dL and men with a hemoglobin of <13 g/dL. The outcomes, assessed on an intention-to-treat basis, included the primary end point (recurrent hospitalization for heart failure and cardiovascular death) and other key secondary end points undertaken in the main trial analysis and ordered as per the original statistical analysis plan^8^: hospitalization for heart failure (recurrent events), cardiovascular hospitalization (first event); cardiovascular death or hospitalization for heart failure (first event); cardiovascular death; cardiovascular death or hospitalization for stroke, myocardial infarction, or heart failure (first event); all-cause mortality; and all-cause mortality or all-cause unplanned hospitalization (first event). Quality-of-life scores were also evaluated using the overall score and physical domain of MLHFQ at 4 and 20 months, because these were the areas of most interest in the original analysis.^8^

Given that a large portion of the trial occurred during the COVID-19 pandemic, in keeping with regulatory guidance,^9^, ^10^, ^11^ a prespecified COVID-19 sensitivity analysis was performed. Here, we present the subgroup results of IRONMAN for the main outcomes for only those patients who were anemic at randomization.

### Statistical Analysis

All formal analyses included treatment group and recruitment context as covariates. Efficacy analyses were done in the intention-to-treat population, excluding 1 patient who was randomized inappropriately. The analyses in this subpopulation were not prespecified and any *P* values quoted should be considered as exploratory. Recurrent events were analyzed by the method of Lin et al^12^ with treatment effects estimated in the form of rate ratios and 95% CIs. Mean frequency functions are displayed using the method of Ghosh and Lin.^13^ Time-to-first-event outcomes were analyzed using Cox proportional hazards models and hazard ratios, 95% CIs, and *P* values calculated. Data are displayed graphically using cumulative incidence functions or Kaplan–Meier curves as appropriate. MLHFQ scores at 4 and 20 months were analyzed using analysis of covariance, adjusting for the randomization stratification variable. Patients who died were assigned the worst possible score after death. Multiple imputation methods were used to account for other missing scores within each treatment group separately using SAS PROC MI using the regression approach adjusting for the stratification variable. Fifty datasets were generated and results analyzed by analysis of covariance within each dataset and results combined using Rubin's rules using the SAS PROC MIANALYZE procedure.

Safety analyses were done for patients assigned to FDI who received ≥1 infusion and all patients assigned to usual care. Proportions of patients having serious adverse events in each system organ class were compared between treatment groups assuming binomial distributions.

We conducted a prespecified COVID-19 sensitivity analysis including only patients randomized until March 31, 2020, around the start of the first national lockdown in the UK. The censoring date was September 30, 2020, based on the assumption that most patients would remain iron replete for ≥6 months from their last dose of FDI or last test showing iron repletion.

All analyses used SAS version 9.4 or R version 3.6.1.

## Results

Of 1137 patients randomized, 771 (68%) were anemic at baseline and were randomly assigned to receive either IV FDI (*n* = 397) or usual care (*n* = 374). The median duration of follow-up was 2.6 years (interquartile range [IQR] 1.4–3.5). Baseline patient characteristics and cardiovascular medications were similar for randomized groups (Table 1). The median age of the patients was 74 years (IQR 68–80 years) and 602 (78%) were men. Most patients were recruited from outpatient clinics (*n* = 502; 65%) with a raised plasma N-terminal pro-brain natriuretic peptide or brain natriuretic peptide. A further 130 patients (17%) were enrolled during a hospital admission for heart failure and 139 (18%) had a prior heart failure admission within 6 months. The baseline median (IQR) hemoglobin was 11.5 g/dL (10.8–12.2 g/dL), serum ferritin was 48 µg/L (IQR 28–86 µg/L), and TSAT was 14% (IQR 9%–18%).

**Table 1:** Baseline Characteristics of Patients With Anemia at Randomization

Characteristic	Ferric Derisomaltose (*n* = 397)	Usual Care (*n* = 374)
Age, years	74 (68–81)	74 (68–80)
Male sex	319 (80)	283 (76)
Body mass index, kg/m^2^	29 (25–33)	28 (25–33)
Race		
White	356 (90)	343 (92)
Black	10 (3)	5 (1)
Asian	28 (7)	22 (6)
Other	3 (1)	4 (1)
Recruitment context		
Heart failure predischarge	66 (17)	64 (17)
Recent (within 6 months) heart failure in-patient	69 (17)	70 (19)
Outpatient with raised natriuretic peptide level	262 (66)	240 (64)
NYHA functional classification		
II	213 (54)	194 (52)
III	175 (44)	174 (47)
IV	9 (2)	6 (2)
Heart rate, beats/min	70 (60–80)	69 (61–80)
Systolic blood pressure, mm Hg	118 (105–132)	118 (105–132)
Left ventricular ejection fraction, %	31 (25–37)	35 (26–37)
Principal cause of heart failure		
Ischemic	233 (59)	215 (58)
Nonischemic	121 (31)	122 (33)
Unknown	43 (11)	37 (10)
Prior medical history		
Hospitalization for heart failure	238 (60)	222 (59)
De novo hospitalization for heart failure	35 (9)	39 (10)
Atrial fibrillation	201 (51)	170 (46)
Acute coronary syndrome	206 (52)	197 (53)
Hypertension	214 (54)	212 (57)
Diabetes mellitus§	191 (48)	197 (53)
Device therapy		
Implantable cardioverter–defibrillator^¶^	54 (14)	49 (13)
Cardiac resynchronization therapy^‖^	95 (24)	77 (21)
Hemoglobin, g/dL	11.6 (10.8–12.3)	11.4 (10.8–12.1)
TSAT, %	14 (10–18)	14 (9–19)
Ferritin, µg/L	49 (28–88)	47 (28–82)
TSAT <20%	307 (77)	296 (79)
Estimated glomerular filtration rate (calculated by CKD EPI), mL/min/1.73 m2	50 (37–67)	49 (36–67)
Heart failure medication		
Loop diuretic	328 (83)	317 (85)
Angiotensin-converting enzyme inhibitor	195 (49)	185 (50)
Angiotensin receptor blocker	58 (15)	72 (19)
Sacubitril–valsartan	83 (21)	69 (18)
Angiotensin-converting enzyme inhibitor, angiotensin receptor blocker, or sacubitril–valsartan	331 (83)	322 (86)
Beta-blocker	349 (88)	332 (89)
Mineralocorticoid receptor antagonist	227 (57)	205 (55)
Digoxin	48 (12)	46 (12)
Sodium-glucose cotransporter-2 inhibitor	13 (3)	8 (2)

NYHA, New York Heart Association; TSAT, transferrin saturation.

Values are median (interquartile range) or number (%).

### Iron Treatment

Of the 397 patients randomized to IV FDI, 391 (98%) received ≥1 dose: 149 patients (38%) received only 1 infusion, 155 (40%) received 2 infusions, 59 (15%) received 3 infusions, and 28 (7%) received between 4 and 9 infusions. Among the 374 participants assigned to usual care, 79 (21%) received IV iron off protocol, with 55 (15%) receiving 1 infusion, 19 (5%) receiving 2 infusions, and 5 (1%) receiving between 3 and 5 infusions.

### Primary and Secondary End Points

**Fig. 1:**
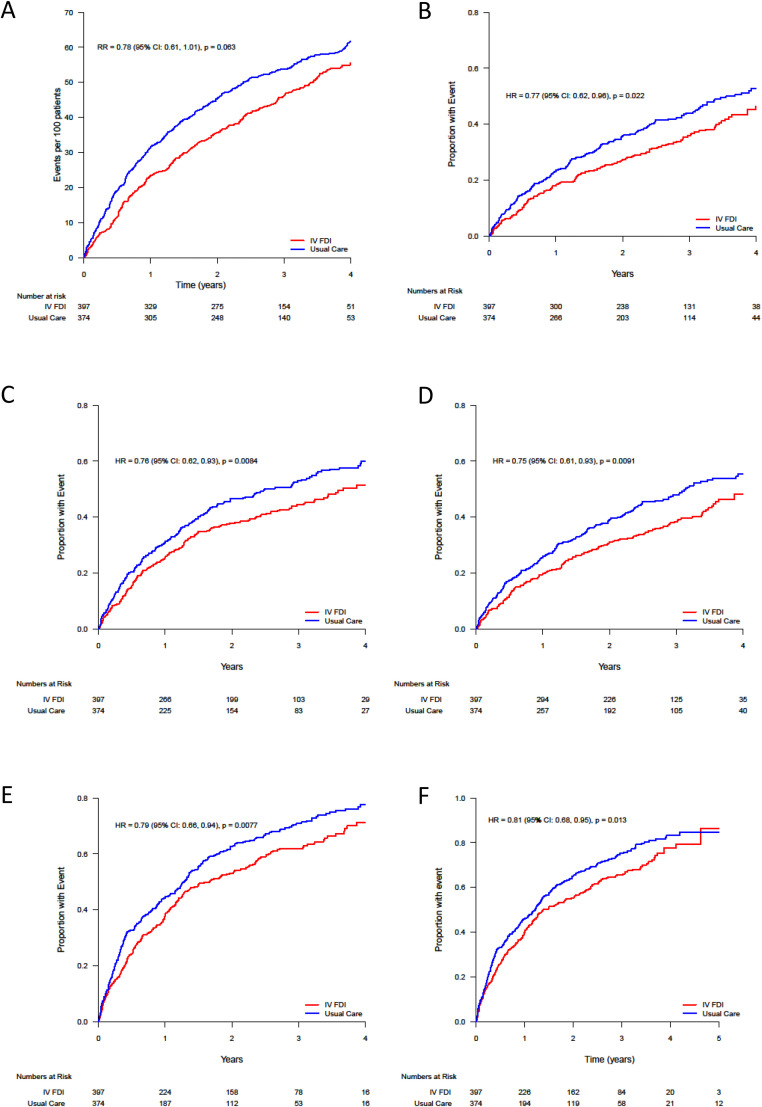
Estimated mean frequency functions and cumulative incidence curves for key cardiovascular outcomes. (A) Cumulative events for the primary efficacy end point (cardiovascular death and hospitalizations for heart failure). Recurrent events plotted in the form of mean frequency functions. (B) Cardiovascular death or hospitalization for heart failure (first event). (C) Cardiovascular hospitalization (first event). (D) Cardiovascular death or hospitalization for stroke, myocardial infarction, or heart failure. (E) All-cause hospitalization (first event). (F) All-cause mortality or all-cause unplanned hospitalization (first event). B, C, D, E, and F show cumulative incidence functions, correcting for the competing risk of noncardiovascular death. The hazard ratios (HR) and rate ratios (RR) are with 95% confidence intervals and were adjusted for the baseline stratification variable of recruitment context (in hospital for heart failure, recent hospital admission for heart failure (within 6 months), or with elevated natriuretic peptide level.

A total of 253 primary end points (25.0 per 100 patient-years) occurred in patients randomized to FDI, compared with 304 end points (32.4 per 100 patient-years) in those randomized to usual care, resulting in a rate ratio of 0.78 (95% CI 0.61–1.01; *P* = .063) (Table 2, Fig. 1). In general, secondary clinical outcomes favored patients assigned to FDI (Table 2, Fig. 1). When considering the individual components of the primary end point, although there were numerically fewer heart failure hospitalizations and cardiovascular deaths with FDI, neither analysis reached statistical significance. Cardiovascular hospitalization as a first event was lower and statistically significant in the group receiving FDI compared with usual care (hazard ratio 0.76, 95% CI 0.62–0.93, *P* = .0084), as was the time to first event of hospitalization for heart failure or cardiovascular death (hazard ratio 0.77, 95% CI 0.62–0.96, *P* = .022), and the time to first event for cardiovascular death or hospitalization for myocardial infarction, stroke or heart failure (hazard ratio 0.75, 95% CI 0.61 to 0.93, *P* = .0091).

**Table 2:** Primary and Secondary End Points for Patients With Anemia at Randomization

End Point	Ferric Derisomaltose (*n* = 397)	Usual Care (*n* = 374)	Estimated Treatment Effect (95% CI)	*P* Value
Primary end point				
Cardiovascular death and hospitalization for heart failure — no. of events (rate per 100 patient- year)	253 (25)	304 (32)	0.78 (0.61 to 1.01)*	0.063
Secondary end points				
Hospitalizations for heart failure — no. of events (rate per 100 patient-year)	189 (19)	227 (24)	0.78 (0.58 to 1.07)*	0.12
Cardiovascular hospitalization (first event)	177 (45)	202 (54)	0.76 (0.62 to 0.93)†	0.0084
Cardiovascular death or hospitalization for heart failure (first event)	146 (37)	172 (46)	0.77 (0.62 to 0.96)†	0.022
Cardiovascular death	90 (23)	109 (29)	0.77 (0.58 to 1.02)†	0.068
Cardiovascular death or hospitalization for stroke, myocardial infarction, or heart failure (first event), *n* (%)	155 (39)	184 (49)	0.75 (0.61 to 0.93)†	0.0091
All-cause mortality	134 (34)	150 (40)	0.83 (0.66 to 1.05)†	0.13
All-cause hospitalization (first event)	246 (62)	264 (71)	0.79 (0.66 to 0.94)†	0.0077
All-cause mortality or all-cause unplanned hospitalization (first event), *n* (%)	262 (66)	280 (75)	0.81 (0.68 to 0.95)†	0.013
Physical domain of Minnesota Living With Heart Failure Questionnaire at 4 months, least squares mean (SE)	18 (1)	21 (1)	–2.94 (–4.68 to –1.20)‡	0.00093
Physical domain of Minnesota Living With Heart Failure Questionnaire at 20 months, least squares mean (SE)	20 (1)	22 (1)	–1.90 (–4.07 to 0.26)	0.084
Overall score of Minnesota Living With Heart Failure questionnaire at 4 months, least squares mean (SE)	37 (1)	42 (2)	–5.17 (–9.25 to –1.09)‡	0.013
Overall score of Minnesota Living With Heart Failure Questionnaire at 20 months, least squares mean (SE)	42 (2)	46 (2)	–4.22 (–9.31 to 0.86)	0.10

SE, standard error.

⁎ Rate ratio.

† Hazard ratio.

‡ Estimated mean difference using multiple imputation and Rubin's rules .

Although no statistically significant between treatment difference was seen for all-cause mortality, FDI was associated with a statistically significant reduction in the outcomes of all-cause hospitalization and all-cause mortality or all-cause unplanned hospitalization (Table 2).

### Quality of Life

At 4 months, patients randomized to receive FDI had better overall quality of life scores (estimated mean difference –5.17, 95% CI –9.25 to –1.09, *P* = .013) and physical domain (–2.94, 95% CI –4.68 to –1.20, *P* = .00093) scores on the MLHFQ compared with those in the usual care group (Table 2). The absolute differences in scores at 20 months were similar to those at 4 months, but these did not reach statistical significance.

### Hemoglobin and Hematological Parameters

At follow-up visits, TSAT and ferritin measurements were assessed only for patients randomized to FDI. For patients that received at least one dose of FDI, TSAT increased from a median of 14% (IQR 10%–18%) at baseline to 27% (IQR 21%–34%) at 4 weeks and 27% (IQR 21%–32%) at 4 months. Serum ferritin increased from a median of 49 µg/L (IQR 28–88 µg/L) at baseline to 466 µg/L (IQR 331–639 µg/L) at 4 weeks, and then 301 µg/L (IQR 207–424 µg/L) at 4 months. For patients assigned to IV FDI who received ≥1 dose (*n* = 391), hemoglobin levels increased from median 11.6 g/dL (IQR 10.8–12.3 g/dL) at baseline to 12.3 g/dL (11.6–13.0 g/dL) at 4 weeks, and to 12.5 g/dL (11.7–13.5 g/dL) at 4 months. For patients assigned to usual care (*n* = 374) the median values were 11.4 g/dL (IQR 10.8–12.1 g/dL) at randomization, 11.6 g/dL (IQR 10.9–12.4 g/dL) at 4 weeks, and 11.9 g/dL (IQR 10.9–12.7 g/dL) at 4 months.

### Serious Adverse Events

Patients assigned to FDI experienced significantly fewer serious adverse events (Table 3), primarily driven by fewer heart failure, arrhythmias and acute coronary events, and had no increase in serious adverse events in any system organ class as defined in the Medical Dictionary for Regulatory Activities (Table 3). Of note, FDI did not increase the number of hospital admissions or deaths owing to infection. For patients assigned to usual care, there were 82 blood transfusion events (8.7 per 100 patient-years) compared with 46 blood transfusion events (4.6 per 100 patient year-) in those assigned to FDI (*P* = .07).

**Table 3:** Serious Adverse Events Tabulated by MedDRA System Organ Class and Preferred Term With 95% CIs for Differences in Percentages

Serious adverse Events by System Organ Class	Ferric Derisomaltose (*n* = 391)	Usual Care (*n* = 374)	Difference (95% CI)	*P* Value
	*n* (%)	*n* (%)		
All	291 (74)	305 (82)	–7.13 (–12.97 to –1.28)	0.017
Cardiac	143 (37)	173 (46)	–9.68 (–16.64 to –2.73	0.0063
Infections and infestations	101 (26)	107 (29)	–2.78 (–9.09 to 3.53)	0.39
Surgical and medical	59 (15)	52 (14)	1.19 (–3.80 to 6.17)	0.64
Gastrointestinal	43 (11)	50 (13)	–2.37 (–7.01 to 2.27)	0.32
Injury, poisoning, and procedural	44 (11)	44 (12)	–0.51 (–5.04 to 4.01)	0.82
Respiratory, thoracic and mediastinal	36 (9)	46 (12)	–3.09 (–7.49 to 1.30)	0.17
Renal and urinary	43 (11)	49 (13)	–2.10 (–6.72 to 2.51)	0.37
General and administration site	43 (11)	36 (10)	1.37 (–2.94 to 5.68)	0.53
Nervous system	36 (9)	32 (9)	0.65 (–3.38 to 4.68)	0.75
Metabolism and nutrition	27 (7)	36 (10)	–2.72 (–6.63 to 1.19)	0.17
Vascular disorders	26 (7)	27 (7)	– 0.57 (–4.17 to 3.03)	0.76
Neoplasms benign, malignant, and unspecified	14 (4)	16 (4)	–0.70 (–3.45 to 2.06)	0.62
Musculoskeletal and connective tissue	12 (3)	22 (6)	–2.81 (–5.75 to 0.12)	0.06
Prespecified safety end points				
Death owing to infection	24 (6)	21 (6)	1.09 (0.61 to 1.95)*	0.78
Hospitalizations owing to infection (first event)	79 (20)	93 (25)	0.77 (0.57 to 1.04)*	0.091

CI, confidence interval.

Counts are number of patients with at least one event in each category. Prespecified infection related safety end points are also shown.

⁎ Hazard ratio (estimated using a Cox proportional hazards model). †Rate ratio (estimated using a negative binomial regression model).

### COVID-19 Sensitivity Analysis

The predefined COVID-19 sensitivity analyses included 92% of all randomly assigned patients (363 receiving FDI and 350 receiving usual care). The rate ratio for the primary outcome was 0.73 (95% CI 0.53–1.01) and not statistically significant (*P* = .054) (Table S1), and secondary clinical outcomes again favored patients assigned to FDI.

## Discussion

This analysis, evaluating the impact of repeated dosing with IV FDI on patients with anemia, iron deficiency, and heart failure with a reduced LVEF, found a reduction in the primary end point of recurrent heart failure hospitalization and cardiovascular death. Although this difference did not reach statistical significance, it was of similar magnitude to that observed in the main IRONMAN trial^8^ and subsequent meta-analyses^4^^,^^14^ and prespecified COVID-19 sensitivity analyses. Of note, when the primary composite end point was analyzed as a time to first event analysis, a stronger effect was identified, possibly because many of these events occurred before the COVID-19 pandemic, when patients assigned to FDI were more likely to be iron replete. In addition, there were statistically significant decreases in the risk of cardiovascular hospitalization (24% lower) and the combined end point of cardiovascular hospitalization or cardiovascular death (25% lower) with FDI. There were numerically fewer cardiovascular deaths in the FDI arm.

For patients with anemia, the IRONMAN results suggest that IV FDI not only decreases cardiovascular end points, but also decreases a broader range of outcomes, including a 21% reduction in all cause hospitalization and a 19% reduction in the composite end point of all-cause hospitalization or all-cause mortality. It is likely that, in patients with heart failure and iron deficiency, those with anemia may be more symptomatic and at greater risk of adverse outcomes. Anemia may also help to identify patients with true iron deficiency. Iron is not just needed for hemoglobin and oxygen carriage and delivery, but has many other essential biological roles. These include generation of cellular energy and mitochondrial function, as well as immunological pathways.^15^, ^16^, ^17^, ^18^ It is plausible that the correction of iron deficiency improves the immune function of patients with heart failure and/or makes them more resilient to the adverse impact of other comorbidities or illnesses. We also found that a greater increase in hemoglobin was seen in patients receiving FDI and this was apparent as early as 4 weeks. Evidence of the specific impact of IV iron on noncardiovascular end points merits further evaluation in future meta-analyses.

In AFFIRM-AHF (A Randomized, Double-blind Placebo-controlled Trial Comparing the Effect of Intravenous Ferric Carboxymaltose on Hospitalizations and Mortality in Iron-deficient Subjects Admitted for Acute Heart Failure), which investigated the effects of IV ferric carboxymaltose on recurrent heart failure hospitalization and cardiovascular death in patients randomized predischarge after heart failure decompensation, 54.6% were anemic at baseline (according to the WHO definition).^2^ In the analysis that included anemic patients, the annualized event rate for the primary outcome with ferric carboxymaltose was 68.9 per 100 patient-years as compared with 81.3 in those patients receiving placebo (rate ratio 0.85, 95% CI 0.61–1.18).^19^ This analysis of the AFFIRM-AHF trial did not report the impact on of ferric carboxymaltose on all-cause hospitalization and death.^19^

There were fewer cardiovascular deaths in this analysis with FDI as compared with usual care alone, although this did not reach statistical significance. The HEART-FID (Randomized Placebo-controlled Treatment for Heart Failure With Iron Deficiency) trial, with >3000 patients with heart failure and reduced ejection fraction recently showed a modest impact of ferric carboxymaltose on the hierarchical end point of all-cause mortality, heart failure hospitalization, and change in 6-minute walk test.^20^ Of note, the mean baseline TSAT was much higher than seen in either IRONMAN or AFFIRM-AHF.^2^^,^^8^ A nonsignificant decrease in cardiovascular death (14%) was seen with IV iron in HEART-FID.^20^ A subsequent individual patient meta-analysis of heart failure trials with ferric carboxymaltose, including HEART-FID, showed a significant decrease in the coprimary end points of cardiovascular hospitalization and cardiovascular death.^21^ Subgroup analyses for this outcome showed a significant interaction with baseline TSAT level and a trend toward interaction with hemoglobin, suggesting that the benefit of IV ferric carboxymaltose was primarily seen in patients with a low TSAT or hemoglobin.^20^ Given these findings, it is timely to review the diagnostic criteria used to identify patients with heart failure who might gain most benefit from IV iron. This work is likely to focus on patients with a reduced TSAT (<20%) and those with a lower hemoglobin. Further individual patient meta-analyses, including data from IRONMAN, will help in this respect and also explore the important question as to whether correction of iron deficiency in patients with heart failure impacts cardiovascular death and whether this factor is influenced by the presence of anemia.

In this analysis, there was a statistically significant benefit on quality of life assessed by the MLHFQ at 4 months in patients assigned to IV FDI both when considering the total score and specifically the physical domain. The magnitude of improvement in QoL at 20 months was similar to that observed at 4 months, although the CIs were wider and statistical significance was lost, likely because many patients did not report their quality of life at 20 months, partly owing to the disruption in patient visits during the COVID-19 pandemic. In addition, underdosing with IV iron in the FDI arm and the use of IV iron and more blood transfusions in the usual care arm may have played a role. It is plausible that the open-label nature of the IRONMAN trial may have influenced quality-of-life assessments in favor of the FDI arm, or against the FDI iron arm because more symptomatic patients may have been more likely to have attended study visits and had have their quality of life assessed. In the masked AFFIRM-AHF trial, improvements in quality of life (Kansas City Cardiomyopathy Questionnaire-12 overall summary score) were seen with IV ferric carboxymaltose at 6, 12, and 24 weeks in the cohort of patients with anemia.^2^ No statistically significant differences were found beyond 24 weeks, a finding that might reflect trial design; no further iron was given beyond this time point.

Over a median follow-up of 2.6 years, it is reassuring that IV FDI was associated with statistically fewer serious adverse events as compared with usual care alone. There was no excess risk of hospitalization or death owing to infection with FDI. Approximately one-fifth of patients assigned to usual care received IV iron outside the protocol; as such, this factor may have decreased the magnitude of benefit of IV FDI in our analyses, which were all performed on an intention-to-treat basis. Given that, in some countries, FDI's labelled indications are restricted to patients with iron deficiency anemia (which includes those with heart failure), these findings will help to inform clinicians and patients during the shared decision-making process around treatment options.

The results of IRONMAN were undeniably impacted by the COVID-19 pandemic. There were substantial periods of time during the pandemic when research patients were not able to be seen in person (or did not want to attend secondary care institutions); as such, it was impossible to do blood tests to assess iron deficiency or deliver IV iron.^8^ This circumstance will have led to undertreatment within the group assigned to IV FDI and together with use of IV iron in the usual care arm outside the protocol will have almost certainly diluted the magnitude of benefit. Although the COVID-19 sensitivity analyses typically suggested greater decreases in the risk of key cardiovascular end points with IV FDI, the lower number of events in this subpopulation resulted in lower statistical power.

There are potential limitations when considering secondary analyses on subpopulations. However, this is still a large cohort (approximately two-thirds of the main trial population) and with a median age of approximately 74 years and with frequent comorbidities that are representative of the demographic of patients seen in routine clinical practice in higher income countries. A minority of patients were women and the vast majority were Caucasian. Randomization in IRONMAN was not stratified by WHO criteria for anemia, so we cannot assume the randomized groups within this subgroup analysis are completely balanced, although it is reassuring that there are no apparent major differences in measured characteristics. Although the prospective randomized open-label blinded-end point design could have impacted behavior for patients (particularly with respect to quality-of-life assessments) and clinicians, the fact that all hospitalizations and deaths were adjudicated blindly is a strength of the trial. Furthermore, it is unlikely when considering the open-label design of the trial that the behavior of patients (or clinicians) with respect to the various end points will have been influenced by the fact that the patients were anemic, including how patients completed quality-of-life questionnaires. The consistent suggestions of benefit with IV FDI across a number of hard secondary end points (cardiovascular and noncardiovascular) and on quality of life (assessed at 4 months) are reassuring. IRONMAN only included patients with heart failure and reduced or mildly reduced LVEF; as such, data should not be extrapolated to those with values of >45%. Few patients in this dataset were receiving an sodium-glucose co-transporters 2 inhibitor at baseline. Given their potential impact on hemoglobin and iron handling,^22^^,^^23^ more data on the interaction between IV iron and this drug class is needed in patients with heart failure. Although we do not compare patients with and without anemia in this analysis, we have previously shown that, when considering the full IRONMAN trial population, there was not a statistically significant interaction for the primary end point between patients without anemia and those with mild or moderate anemia.^8^

## Conclusions

In a broad range of patients with heart failure with a reduced LVEF and iron deficiency anemia, the administration of IV FDI increased hemoglobin, had favorable medium-term effects on quality of life, may have decreased morbidity and mortality, and had no evidence of harm with fewer serious adverse events. Because the use of FDI is restricted to iron deficiency anemia in some countries, this analysis may help to inform clinicians and patients on the value of administration of IV iron to a group of patients with, on average, poorer quality of life and worse outcomes.

Paul R. Kalra

## Appendix Supplementary materials

Image, application 1

Image, application 2
