# Supplementary material for: The Impact of Ferric Derisomaltose on Cardiovascular and Noncardiovascular Events in Patients With Anemia, Iron Deficiency, and Heart Failure With Reduced Ejection Fraction
Source: J Card Fail. 2024 May;30(5):682–90. doi: 10.1016/j.cardfail.2023.10.006 (PMC11096866; doi:10.1016/j.cardfail.2023.10.006)
Supplement: Supplementary file 1 [file mmc1.docx]

**Table S1: Primary and secondary endpoints in the COVID-19 analysis for patients with anemia at randomization (censoring follow-up on 30 September 2020).**

| **End Point** | **Ferric derisomaltose**  **(N= 363)** | **Usual care**  **(N= 350)** | **Estimated Treatment Effect (95% CI)** | **P-value** |
| --- | --- | --- | --- | --- |
| **Primary endpoint** |  |  |  |  |
| Cardiovascular death and hospitalization for heart failure — no. of  events (rate per 100 patient-yr) | 157 (25) | 210 (34) | 0·73 (0.53 to 1.01)* | 0.054 |
| **Secondary endpoints** |  |  |  |  |
| Hospitalizations for heart failure — no· of events (rate per 100 patient-yr) | 120 (19) | 161 (26) | 0.73 (0.50 to 1.06)* | 0.094 |
| Cardiovascular hospitalization (first event) — no. (%) | 121 (33) | 146 (42) | 0.75 (0.59 to 0.96)** | 0.021 |
| Cardiovascular death or hospitalization for heart failure (first event) — no. (%) | 92 (25) | 118 (34) | 0.73 (0.56 to 0.96)** | 0.026 |
| Cardiovascular death — no. (%) | 52 (14) | 68 (19) | 0.74 (0.52 to 1.06)** | 0.10 |
| Cardiovascular death or hospitalization for stroke, myocardial infarction, or heart failure (first event) — no. (%) | 99 (27) | 129 (37) | 0·71 (0.55 to 0.93)** | 0.011 |
| All-cause mortality — no. (%) | 79 (22) | 91 (26) | 0.84 (0·70 to 1·19)** | 0.27 |
| All-cause hospitalization (first event) — no. (%) | 184 (51) | 205 (59) | 0.80 (0.65 to 0.97)** | 0.026 |
| All-cause mortality or all cause unplanned hospitalisation — no. (%) | 271 (51·4) | 303 (56·5) | 0·89 (0·75 to 1·04)** | 0.15 |

Footnote: SE= standard error, * Rate ratio, ** Hazard ratio.
